# Are TP53 Arg72Pro and MDM2 T309G polymorphisms associated with bladder cancer risk? A meta-analysis

**DOI:** 10.4314/ahs.v24i3.16

**Published:** 2024-09

**Authors:** Rym-Khadidja Abderrahmane, Zohra Touala-Chaila, Khedidja Benseddik, Nihed Hassani, Hind Drider, Imene Derbouz-Draoua, Djebaria Naima Meroufel

**Affiliations:** 1 Université des Sciences et de la Technologie d'Oran Mohamed-Boudiaf USTOMB El Mnaouar, BP 1505, Bir El Djir 31000, Oran, Algérie; 2 Laboratoire de la Génétique Moléculaire et Cellulaire (LGMC) USTOMB El Mnaouar, BP 1505, Bir El Djir 31000, Oran, Algérie; 3 École Supérieure en Sciences Biologiques d'Oran (ESSB- Oran, Algerie) Cité du Chercheur (Ex- IAP), Essenia -Oran. Boîte Postale 1042, Saim Mohamed 31003 Oran Algérie

**Keywords:** Bladder cancer (BC), polymorphism, Meta-analysis, T309G, Arg72Pro, TP53, MDM2

## Abstract

**Background:**

We still do not know the exact cause of bladder cancer (BC).

**Objectives:**

Evaluation of the effect of TP53 Arg72Pro and MDM2 T309G polymorphisms with the risk of Bladder cancer.

**Methods:**

A literature search was conducted followed by a meta-analysis. Then, sensitivity and subgroup analyses were performed. 14 relevant studies were included in the quantitative analysis.

**Results:**

No statistically significant associations were found. The results of the subgroup analysis revealed a significant association in the Turkish population for T309G: G vs. T (P-value= 0.015; OR 95%CI= 1.51 [1.084; 2.125]), GG vs.TT (P-value= 0.009; OR 95% CI= 2.60 [1.262; 5.370]). Sensitivity analysis revealed a significant association between the Arg72Pro: C vs. G (OR= 1.22, 95% CI [1.05; 1.40]), CC vs. GG (OR= 1.54; 95% CI [1.13; 2.09]), CC+CG vs. GG (OR= 1.24; 95% CI [1.01; 1.53]), CC vs. CG+GG (OR= 1.33; 95% CI [1.01; 1.74]), and T309G: G vs. T (OR= 1.30; 95% CI [1.07; 1.57]), GG vs. GT+TT (OR= 1.53; 95% CI [1.10; 2.11]), GG vs. GT (OR=1.44; 95% CI [1.02; 2.02]), GG vs. TT (OR= 1.88; 95% CI [1.25; 2.82]) with BC occurrence.

**Conclusion:**

The T309G polymorphism was found to be a predisposing allele for BC in Turkish population.

## Introduction

Bladder cancer (BC) is a general term for neoplasms affecting the bladder, the most common type being Urothelial Cell Carcinoma (UCC) or Transitional Cell Carcinoma (TCC), which accounts for over 90% of BC cases^1^. Bladder cancer is one of the ten most common types of cancer worldwide and accounts for approximately 550,000 new cases per year. In terms of geographical distribution, countries in Southern and Eastern Europe, Africa, the Middle East, and North America are the most affected by this type of cancer 2. Cigarette smoking and diabetes are the most important risk factors leading to bladder cancer^3^. Genetically, bladder cancer is a polygenic disease with a tendency to have high mutation rates^4^. Because tumor heterogeneity presents a barrier to diagnosis and treatment of bladder cancer, it is very important for research to characterize tumor heterogeneity by integrating genetic and epigenetic features^5^. The tumor suppressor gene TP53 is a major actor in the regulation of cell division and apoptosis induction. Indeed, the p53 protein is located in the nucleus of the organism's cells, where it binds directly to the DNA. When a cell's DNA is damaged, this protein plays a key role in determining whether the DNA will be repaired or whether the damaged cell will self-destruct through apoptosis^6^. Mutations in the TP53 gene are frequently found in human cancers, including bladder cancer. Previous studies have shown mutation of TP53 in nearly half of the Muscle Invasive Bladder Cancer (MIBC) samples and inactivation of TP53 function in 76% of the samples^7^.

The proto-oncogene Mouse Double Minute 2 (MDM2) is the negative regulator of the TP53 gene. Its over-expression has been found in a number of malignant tumors, indicating that this oncogene plays a key role in human carcinogenesis^8^. It binds to and degrades the p53 protein, leading to decreased levels of this key tumor suppressor molecule. Thus, dysregulation of the MDM2 protein can not only induce cell proliferation, but also inhibit apoptosis, promoting cancer occurrence^9^-^10^.

The presence of a single nucleotide polymorphism (SNP) T309G (rs2279744) in the promoter region of MDM2 would result in higher levels of MDM2 protein expression, thus attenuating the p53 pathway. These mechanisms would increase the susceptibility to develop certain types of cancer. The TP53 gene also has a functional polymorphism, the G:C transition at codon 72 which results in the substitution of an Arg amino acid into Pro. It appears that the Arg/Arg genotype induces apoptosis with faster kinetics and inhibits transformation more effectively than the Pro/Pro genotype^11^.

Based on these findings, we attempt to quantitatively assess the association between the Arg72Pro (rs1042522) polymorphisms of the TP53 gene and T309G of the MDM2 gene with the susceptibility to developing bladder cancer.

## Methods

### Research strategy

A literature search was performed on PubMed and Google Scholar databases until 04/03/2022 in order to identify the different studies that analyzed the association of TP53 Arg72Pro and MDM2 T309G polymorphisms with bladder cancer using the following terms and keywords: (Bladder cancer) AND (Arg72Pro), ((Bladder cancer) AND (TP53)) AND (Arg72Pro), ((Urothelial carcinoma) AND (TP53)) AND (Arg72Pro), ((Transitional cell carcinoma) AND (TP53)) AND (Arg72Pro). English was used as a search language. Appendix A shows the research strategy for the MDM2 T309G on PubMed.

### Inclusion and Exclusion criteria

**Inclusion criteria:** Studies assessing the association between the SNPs Arg72Pro of the TP53 gene and T309G of the MDM2 gene with the occurrence of bladder cancer; case-control studies; and availability of study data.

**Exclusion criteria:** duplicate studies, absence of full text, not a case-control study, study not related to TP53 and MDM2 genes or Arg72Pro and T309G polymorphisms, study not related to bladder cancer.

### Data extraction

To ensure consistency and credibility of the data collected, three authors (Drider, Hassani and Derbouz-Draoua) independently extracted the characteristics of each of the included studies: name of the author, study population, genotyping methods as well as the number of individuals per genotype. To ensure accuracy, discrepancies were discussed with two other authors (Touala-Chaila and Abderrahmane) until a final validation was obtained.

### Data analysis

In this quantitative study, all statistical calculations were performed using MetaGenyo (12), a web-based tool available online at https://metagenyo.genyo.es/. The Hardy-Weinberg equilibrium (HWE) test was recalculated in control. The study was considered to be in HWE if both the p values and adjusted p values were greater than 0.05. The determination of ORs (95% CI) and p values for the meta-analysis of the Arg72Pro and T309G polymorphisms that correspond to the TP53 and MDM2 genes, respectively, was performed by applying the statistical methods Inverse Variance (for the fixed-effects analysis model) and DerSimonian-Laird (for the random-effects analysis model) , where a p value of less than 0.05 was considered to be statistically significant. A subgroup and sensitivity analysis were performed in order to explain the source of the heterogeneity. The heterogeneity analysis was conducted by calculating the I2 and the p-value (phet) if p < 0.1 this indicates that there is significant heterogeneity between the data being used in the quantitative analysis. The choice of the random effect model (REM) or the fixed effect model (FEM) will be automatically made according to the result of the heterogeneity tet.

## Results

### Selection process and the characteristics of the studies

**Fig. 1:**
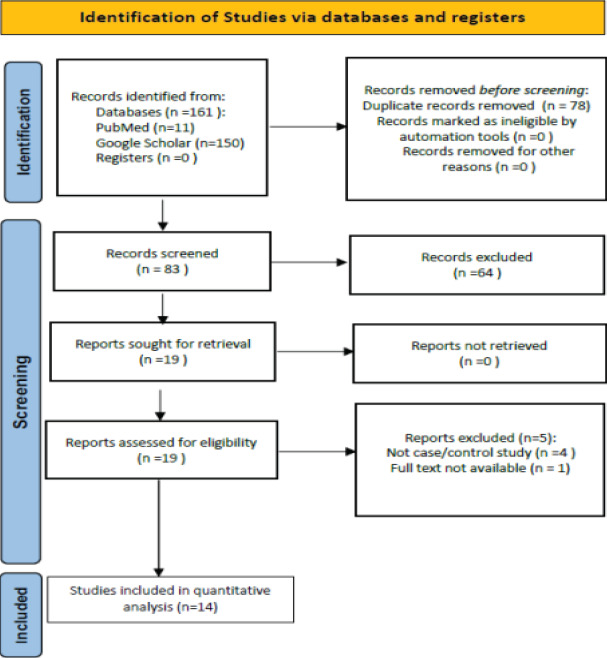
Study selection flow chart

A total of 161 records were identified from the databases searched. After eliminating duplicates, 83 records were retained. According to the content of the abstracts, 64 publications were excluded because 8 of them were reviews or meta-analyses, 47 were not related to bladder cancer, 4 were not related to our genes of interest (TP53/MDM2), 2 did not evaluate polymorphisms of interest, and 3 were not case-control studies. Therefore, 19 studies were obtained, of which 5 were excluded because 1 lacked the full text and the other 4 were not case/control studies. Finally, 14 studies were included in this meta-analysis^13^-^26^. The process of selection according to the “PRISMA 2020 flowchart for new systematic reviews that include only database and registry searches” is illustrated in Figure 1.^27^. Table 1 summarizes the key characteristics of each of the 14 inclued studies.

**Table 1:** main characteristics of the 14 studies included in the quantitative analysis

Study	Population	Geographic region	Génotyping methods	Gene	SNPs	Cases/controls	HWE in controls	
							*P*-value*	Adjusted *P*-value**
**19.Berrada et al.2013**	Moroccan	Northen Africa	Allele-specific PCR	***TP53***	**rs1042522**	41/38	0,1002	0,188
**17.Pineda et al. 2014**	Spanish	Southern Europe	Illumina Golden Gate and TaqMan assays			1032/1100	0,0282	0,118
**20.Törüner et al. 2001**	Turkish	Southeastern Europe, Western Asia	PCR and Restriction Digestion **(PCR-RFLP)**.			121/114	0,884	0,884
**21.Arshad et al.2010**	Kashmiri	South-Central Asia	PCR-RFLP			108/138	0,0295	0,118
**22.Castro Santos et al.2011**	Brazilian	South America	PCR-RFLP			94/159	0,8084	0,884
**23.Hosen et al.2015**	Bangladeshi	South Asia	PCR-RFLP			102/140	0,1038	0,188
**24.Ronggui et al.2011**	Chinese	Eastern Asia	PCR-RFLP			120/120	0,141	0,188
**26.Yegin et al. 2019**	Turkish	Southeastern Europe, Western Asia	PCR-RFLP			180/163	0,1197	0,188
**13.Yenilmez et al. 2017**	Turkish	Southeastern Europe, Western Asia	PCR-RFLP	***MDM2***	**rs2279744**	40/75	0,9863	0,9967
**14.Avirmed et al. 2017**	Mangolian	Eastern Asia	PCR-RFLP			63/79	0,3105	0,621
**15.Jawad et al. 2018**	Iraq	Middle East,Western Asia	PCR-RFLP			60/40	0,9967	0,9967
**16.Gangwar et al.2010**	North Indian	South-Central Asia	PCR-RFLP			212/250	0,1391	0,621
**18.Hitzenbichler et al. 2014**	Germany (Caucasian)	Western Europe	PCR-RFLP			224/140	0,5142	0,7713
**25.ONAT et al. 2006**	Turkish	Southeastern Europe, Western Asia	PCR/Restriction Digestion **(PCR-RFLP)**			75/103	0,2156	0,621

### Meta analysis and Heterogeneity

**Fig. 2:**
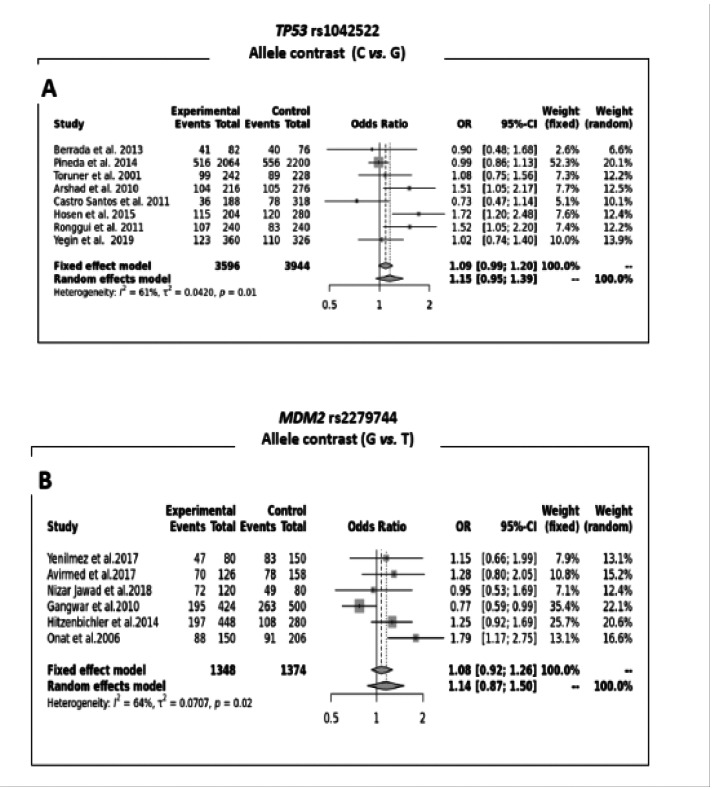
Forest-plot for *TP53/MDM2* genes polymorphisms under the allele contrast genetic model [**A**: *TP53* rs1042522 (C vs. G); **B**: *MDM2* rs2279744 (G vs. T)]

The results of the association tests between rs1042522/rs2279744 polymorphisms of TP53/MDM2 genes with the occurrence of bladder cancer under different genetic models: allele contrast, recessive, dominant, as well as homozygous and heterozygous models are represented in Table 2. Also included in the same table are the results of the heterogeneity tests (Phet and I2 values). The genetic models selected revealed no statistically significant association between the SNPs rs1042522/rs2279744 with the occurrence of bladder cancer. Fig. 2 illustrates the results of the 2 SNPs under the allele contrast genetic model.

**Table 2:** A meta-analysis of the association between rs1042522/rs2279744 polymorphisms of the *TP53/MDM2* genes with BC development

Genetic Models	Association Tests			Heterogeneity Tests			Publication Bias Test
	OR	95% CI	P-value	*Effect Model	*P_het_*-value	I^2^	**Egger's Test *P*-value
***TP53* rs1042522**							
C vs. G ^a^	1.150	[0.951; 1.392]	0.148	REM	0.011	0.613	0.425
CC vs. CG+GG ^b^	1.198	[0.880; 1.631]	0.249	REM	0.082	0.444	0.553
CC+CG vs. GG ^c^	1.212	[0.896; 1.639]	0.210	REM	0.002	0.685	0.417
CG vs. CC+GG ^d^	1.048	[0.777; 1.413]	0.758	REM	0.001	0.706	0.953
CC vs. GG ^e^	1.345	[0.958; 1.888]	0.086	REM	0.089	0.433	0.310
CC vs. CG ^f^	1.118	[0.785; 1.592]	0.535	REM	0.041	0.520	0.595
CG vs. GG ^g^	1.170	[0.842; 1.625]	0.348	REM	0.001	0.700	0.596
***MDM2* rs2279744**							
G vs. T ^a^	1.143	[0.869; 1.503]	0.338	REM	0.017	0.636	0.344
GG vs. GT+TT ^b^	1.267	[0.816; 1.968]	0.290	REM	0.020	0.624	0.454
GG+ GT vs. TT ^c^	1.021	[0.796; 1.311]	0.864	**FEM**	**0.130**	**0.412**	0.110
GT vs. GG+TT ^d^	0.897	[0.722; 1.115]	0.328	**FEM**	**0.491**	**0**	0.264
GG vs.TT ^e^	1.433	[0.808; 2.540]	0.218	REM	0.017	0.637	0.215
GG vs. GT ^f^	1.245	[0.833; 1.862]	0.284	REM	0.075	0.499	0.763
GT vs. TT ^g^	0.960	[0.736; 1.253]	0.766	**FEM**	**0.369**	**0.072**	**0.036**

The studies included in the present meta-analysis, show significant heterogeneity (P < 0.1) for rs1042522 and rs2279744 under all genetic models, except for rs2279744 of the MDM2 gene (dominant [GG+ GT vs. TT (Phet= 0.130; I2= 41%)], Overdominant [GT vs. GG+TT (Phet=0.491; I2= 0%)] and the Heterozygote genetic model [GT vs. TT (Phet= 0.369; I2= 7%)]) where the absence of heterogeneity is largely noticed.

### Sensitivity and subgroup analysis

rs1042522 showed obvious heterogeneity across all genetic models, including C vs. G (Phet= 0.011; I2= 61%), CC vs. CG+GG (Phet= 0.082; I2= 44%), and CC+CG vs. GG (Phet= 0.002; I2= 69%). Heterogeneity was also found in rs2279744 among some of the genetic models: G vs. T ( Phet=0.017; I2= 64%), GG vs. GT+TT (Phet= 0.020; I2= 62%).

**Fig 3:**
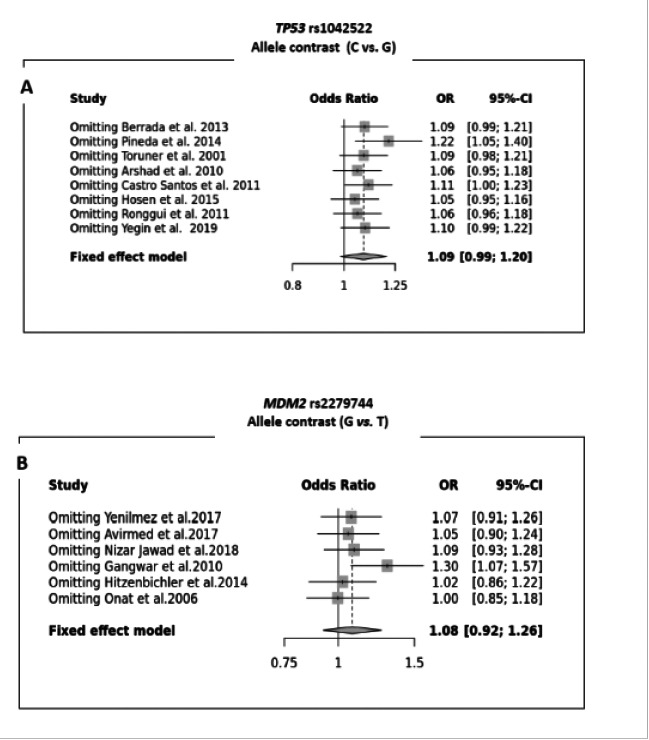
Sensitivity Plot (leave-1-out forest plot) for *TP53/MDM2* genes polymorphisms under the allele contrast genetic model [**A**: *TP53* rs1042522 (C vs. G); **B**: *MDM2* rs2279744 (G vs. T)]

A sensitivity analysis was performed to determine if there were specific studies that had a major effect on the pooled OR results. For rs1042522, the heterogeneity was mainly caused by the Pineda et al. study^17^. When the latter was removed, we discovered a statistically significant association between the SNP Arg72Pro of the TP53 gene and bladder cancer occurrence: C vs. G (OR = 1.22, 95% CI [1.05; 1.40]), CC vs. GG (OR = 1.54; 95% CI [1.13; 2.09]), CC+CG vs. GG (OR = 1.24; 95% CI [1.01; 1.53]), CC vs. CG+GG (OR= 1.33 ; 95% CI [1.01 ; 1.74]). For the T309G polymorphism of the MDM2 gene, sensitivity analysis revealed a significant association between SNP rs2279744 and bladder cancer occurrence when the study of Gangwar et al. 2010 was removed: G vs. T (OR= 1.30; 95% CI [1.07; 1.57]), GG vs. GT+TT (OR= 1.53; 95% CI [1.10; 2.11]), GG vs. GT (OR=1.44; 95% CI [1.02; 2.02]), GG vs. TT (OR= 1.88; 95% CI [1.25; 2.82]). Figure 3 shows the sensitivity plot of the two SNPs under the allelic contrast genetic model.

Two factors were selected for subgroup analysis: geographic regions and genotyping methods (supplementary table 1). Because of the limited number of studies from the same geographic region (<2), no statistically significant association between the rs1042522 of the TP53 gene with BC occurrence was considered. In contrast to rs2279744 of the MDM2 gene, where a statistically significant association between the above SNP and the development of bladder cancer was found in the Southeastern European and Western Asian regions (Turkey) under the genetic models: G vs. T (P-value= 0.015; OR 95%CI= 1.51 [1.084; 2.125]), GG vs. TT (P-value= 0.009; OR 95% CI= 2.60 [1.262; 5.370]).

The genotyping technique PCR-RFLP was found to be associated with the diagnosis of bladder cancer when it was applied to rs1042522 of the TP53 gene: Recessive model CC vs. CG+GG: (P value= 0.025; OR 95% CI= 1.37 [1.038; 1.815]); CC vs.GG (P value= 0.003; OR 95% CI= 1.588 [1.163; 2.167]). However, no genotyping method was found to be associated with the diagnosis of BC when the rs2279744 of the MDM2 gene was concerned.

### Publication bias

A funnel plot should be symmetrical and look like an inverted funnel in the absence of small study effects (publication bias), and thus the dispersion is due to sampling variation alone. Publication bias is a factor that can lead to asymmetry in a funnel plot 28. The main limitation of the funnel plot is that its visual interpretation remains highly subjective (supplementary figure 1). For this reason, a statistical test is used, the Egger's test (Table 2). According to the latter, no publication bias was detected among the studies included in the present meta-analysis, except for (rs2279744) which shows a publication bias under the GT vs. TT genetic model (Egger's Test P-value= 0.036). This can be explained by chance only since all the adopted genetic models have no publication bias.

## Discussion

A total of 1798/1972 cases and controls for the Arg 72 Pro SNP of the TP53 gene and 674/687 for the T309G of the MDM2 gene were obtained from the literature search to assess their associations with BC occurrence. No statistically significant associations were found in the quantitative analysis, except for the MDM2 rs2279744 polymorphism, which showed a significant association with the development of bladder cancer in the Turkish population under the genetic models [G vs. T and GG vs. TT] when using the Subgroup analysis test.

The PCR-RFLP genotyping method was found to be the most frequently used due to the relevance of the conclusion drawn regarding TP53 rs1042522 and the cancer studied [CC vs GG]. This technique has several advantages: it is easy to perform; it does not require expensive equipment; and it does not require extensive training of laboratory personnel. On the other hand, the disadvantages of this technique are as follows: It requires that a variation generate or abolish a restriction enzyme recognition site. Some restriction enzymes are costly. and time-consuming technique^29^.

Note that nonsignificant results (p value > 0.05) from the subgroup analysis are not shown in Supplementary table 1.

The sensitivity analysis revealed that the Arg72Pro of the TP53 gene and the T309G of the MDM2 gene had a deleterious effect on bladder cancer (Fig 3) for the rs1042522 of the TP53 gene, its association is in agreement with the meta-analyses conducted on Asian populations that were performed by Liu et al^30^, Yang et al (31), and Zhang et al (32), and in discordance with the work of^33^ performed on Caucasian, where no association was found. This inconsistency could be explained by the existence of additional genetic and environmental factors. In fact, P53 is considered to be the guardian of the genome and plays a specificole in the malignant transformation of normal cells. If mutations occur in P53, its function is altered, leading to the development of malignant cells or even cancerous disease^34^. Different biochemical properties have been reported for the two P53 variants. The P53 Arg 72 version is more efficient at inducing apoptosis, while the P53 Pro 72 version has a greater capacity for DNA repair and more strongly induces cell cycle arrest^35^.

Significant heterogeneity was found in the association test, which may be explained by the presence of false positive results in the original studies or false negative results in the small replication studies. The inconsistency and heterogeneity between studies may be due to bias or to true differences in genetic effects between populations^36^.

In the case of our study, we found that the source of heterogeneity was represented in the diversity of genotyping techniques used and in the geographic distribution of the different populations included in our meta-analysis, because when a subgroup analysis was performed according to these two factors, homogeneity was revealed (FEM as an index of homogeneity) (supplementary table 3).

As for the MDM2 rs2279744, it's association is in agreement with the meta-analysis of Xie et al. where they found that the SNP309 T>G polymorphism is not associated with bladder cancer risk development in Asians, but it may be associated with genetic susceptibility for bladder cancer in Caucasians^37^. And in discordance of the meta-analysis of Ding et al., where no significant association between the T309G and bladder cancer risk susceptibility^38^.

MDM2 is an important negative regulator of the P53 protein. High levels of MDM2 expression decrease P53 protein levels and function, leading to an increased risk of cancer and/or accelerated tumor formation and progression. Most of the increased expression of MDM2 in human tumors is due to the amplification of the gene. On the other hand, SNP309, a naturally occurring single nucleotide polymorphism (SNP) in the MDM2 gene (a G to T change in the regulatory region of intron 1), increases MDM2mRNA expression levels, which correlates with an increased risk of several human cancers^39^.

## Conclusion

In our systematic review, we identified a total of 14 relevant studies, targeting two SNPs of two different genes: TP53 and MDM2. No statistically significant association was found between TP53 rs1042522 and MDM2 rs2279744 with bladder cancer occurrence. However, subgroup analysis revealed a significant association in the South-eastern Europe, Western Asia (Turkey) population for SNP rs2279744. Sensitivity analysis revealed a statistically significant association with a deleterious effect of the two SNPs studied. Further studies in different populations are needed to support the conclusion of the impact of the SNPs selected in the present analysis on BC occurrence.

## Appendix A

((Urothelial carcinoma) AND (MDM2)) AND (T309G)

((Transitional cell carcinoma) AND (MDM2)) AND (T309G)

((Bladder cancer) AND (MDM2)) AND (T309G)
